# Microendoscopic Posterior Decompression for L5–S1 Foraminal and Extraforaminal Lesions: A Comparative Study of Herniation Versus Stenosis

**DOI:** 10.7759/cureus.109559

**Published:** 2026-05-24

**Authors:** Hirohiko Inanami, Ryoji Tominaga, Hiroki Iwai, Hisashi Koga

**Affiliations:** 1 Spine Surgery, Inanami Spine and Joint Hospital, Tokyo, JPN; 2 Spine Surgery, Iwai Orthopaedic Hospital, Tokyo, JPN

**Keywords:** extra-foraminal disc herniation, extraforaminal stenosis, far-out syndrome, lumbosacral junction, microendoscopic decompression surgery, posterior spinal decompression

## Abstract

Introduction

Foraminal and extraforaminal nerve root compression at the lumbosacral junction (L5/S1) is an underrecognized cause of lumbar radiculopathy. The L5 nerve root traverses a narrow space between the transverse process and the sacral ala and may be compressed by migrated disc fragments or fibro-osseous structures. Although lumbar disc herniation (LDH) and foraminal or extraforaminal stenosis (FES) at L5/S1 are often evaluated together, their mechanisms differ. This study aimed to compare postoperative clinical outcomes between patients with L5-S1 foraminal and extraforaminal disc herniation and those with stenosis who underwent microendoscopic posterior decompression.

Materials and methods

This retrospective observational study included consecutive patients who underwent microendoscopic posterior decompression for isolated L5/S1 foraminal or extraforaminal lesions between 2015 and 2019. All procedures were performed by a single surgeon. Patients were classified into the LDH or FES group based on preoperative MRI and CT findings, with final confirmation by intraoperative findings. Patients with previous lumbar surgery at the index level, radiographic instability or spondylolisthesis requiring fusion, and multilevel symptomatic lesions were excluded. Functional outcomes were assessed using the Oswestry Disability Index (ODI), with achievement of the minimal clinically important difference (MCID; ≥13-point improvement) at 12 months as the primary endpoint. Leg and low back pain were assessed using the numeric rating scale (NRS).

Results

Forty patients completed 12-month follow-up (LDH, n = 19; FES, n = 21). ODI improvement was greater in the LDH group than in the FES group (median ΔODI, −24.4 (IQR, −36.0 to −10.9) vs. −17.8 (IQR, −22.2 to −2.2); p = 0.031). MCID achievement rates were comparable between the groups (73.7% in LDH and 61.9% in FES; p = 0.511). Improvements in leg pain and low back pain NRS did not differ significantly between the groups (p = 0.369 and p = 0.201, respectively).

Conclusions

Microendoscopic posterior decompression for L5/S1 foraminal or extraforaminal lesions resulted in similar rates of clinically meaningful improvement in LDH and FES. However, functional recovery was greater in LDH, suggesting that patients with FES may require different postoperative expectations regarding the degree and time course of functional recovery.

## Introduction

Lumbar pathologies are generally categorized as central canal, foraminal, or extraforaminal lesions [1,2]. Among these, foraminal and extraforaminal disorders at the lumbosacral junction (L5/S1) are often overlooked compared with central canal lesions and can cause persistent radiculopathy or residual symptoms postoperatively [3-5]. At the L5/S1 level, the L5 nerve root passes through a narrow space between the transverse process and the sacral ala, known as the lumbosacral tunnel or far-out region, where it is easily affected by mechanical compression [5,6]. Additional factors, such as osteophytes or migrated disc fragments, may further contribute to nerve entrapment. Despite reports describing various surgical techniques and associated outcomes, the underlying pathologies and clinical manifestations remain incompletely understood, underscoring the need for careful evaluation based on the characteristic anatomy of the L5/S1 segment [3,7].

Previous studies analyzed isolated disc herniation-related lesions and extraforaminal stenosis together under the same conditions and also included mixed-level cases attributable to L4/5 pathology [8-10]. This heterogeneity makes it difficult to clarify the differences in clinical presentation and treatment response, especially because lumbar disc herniation (LDH) often presents with acute or subacute compression caused by a herniated disc fragment, whereas foraminal or extraforaminal stenosis (FES) usually reflects chronic stenosis due to fibrous or bony structures [8,9]. Thus, high-quality evidence focusing only on pure L5/S1 foraminal or extraforaminal lesions and separately comparing disc herniation with stenosis remains insufficient [3,5,10].

Therefore, the present study examined whether foraminal or extraforaminal LDH and FES at L5/S1 should be considered distinct clinical entities in terms of treatment response and functional recovery patterns. Although these two pathologies are often evaluated together, their underlying mechanisms are distinct, and their therapeutic responses may likewise differ. To this end, we analyzed consecutive cases treated by a single surgeon at a single institution, focusing only on isolated L5/S1 foraminal or extraforaminal lesions, and compared the clinical features and postoperative improvements between LDH and FES.

## Materials and methods

Setting and study participants

This retrospective observational study analyzed data from patients treated at a single institution by a single surgeon between January 1, 2015, and December 31, 2019. Consecutive patients who underwent posterior microendoscopic decompression or discectomy for L5/S1 foraminal or extraforaminal lesions performed by the same surgeon (H.I.) at Iwai Orthopaedic Hospital and Inanami Spine and Joint Hospital were included. The study protocol was approved by the Institutional Review Board of Iwai Medical Foundation (approval number: 20200608-3).

All patients underwent a detailed neurological examination and standardized magnetic resonance imaging (MRI), including an axial slice through the center of the L5/S1 intervertebral disc. Computed tomography (CT) was performed when required. Based on these imaging findings and clinical presentations, patients with suspected L5 nerve root disorders were screened. Patients who met the inclusion criteria were included in the final analysis.

Inclusion criteria

Eligible patients met the following criteria: first, pain or sensory disturbance corresponding to the L5 dermatome, and selective L5 nerve root block reproduced the symptoms and achieved ≥60% pain relief; second, MRI or CT findings demonstrating lesions in the L5/S1 foraminal or extraforaminal regions; third, eligible patients who completed the 12-month postoperative follow-up. Selective L5 nerve root blocks were performed under fluoroscopic guidance using 1% lidocaine.

Exclusion criteria

Patients were excluded if their L5 nerve root disorder was judged to originate from levels other than L5/S1, or if factors that could compromise analytical homogeneity were present. Specifically, patients with central or foraminal stenosis or disc herniation at L4/5 that could influence the L5 nerve root were excluded. Patients who underwent additional surgery at levels above L4/5 or those with a previous surgical history in the L5/S1 foraminal or extraforaminal area were also excluded. Finally, patients with instability requiring fusion surgery, those with missing preoperative Oswestry Disability Index (ODI) data, and those diagnosed with nonradicular pain originating from the facet joint or sacroiliac joint were also excluded. Instability was assessed preoperatively using flexion-extension lateral radiographs and was defined as >3 mm translational motion or >10° angular change.

Definition of disease classification

Classification into the LDH and FES groups was primarily based on intraoperative findings, with reference to preoperative imaging and clinical information. LDH was defined as a lesion in the far-out or far-extraforaminal region lateral to the pedicle, in which compression of the L5 nerve root by a migrated disc fragment was confirmed, and decompression was completed by removal of disc material. FES was defined as fibrous or osseous stenosis from the foraminal to the extraforaminal outlet zone, appearing on T2-weighted MRI, loss or replacement of fat signals within the region bordered by the intervertebral disc, sacral ala, and superior articular process of S1, with flattening or displacement of the nerve root. Cases in which decompression was completed without disc removal were classified as FES. To avoid pathological heterogeneity and misclassification, borderline cases in which intraoperative findings indicated comparable contributions of the discal mass effect and fibroosseous stenosis, making the dominant pathology indeterminate, were excluded.

Surgical procedure

All surgeries were performed with the patient in the prone position on a radiolucent table. A C-arm fluoroscope was adjusted to overlap the bilateral L5/S1 foramina. A skin incision of approximately 18 mm was made 50-60 mm lateral to the midline of the L5/S1 disc level. After blunt splitting of the paraspinal muscles, sequential dilators were inserted, followed by a tubular retractor. The procedure was performed under micro-endoscopic visualization.

The far-extraforaminal region, comprising the L5 transverse process, pars interarticularis, sacral ala, and lateral margin of the L5-S1 facet joint, was identified. Bleeding in the operative field was controlled using bipolar coagulation and hemostatic agents. Fibrous bands covering the dorsal part of the nerve root, thickened ventral joint capsule, and lateral osteophytes were removed stepwise using Kerrison rongeurs and high-speed drills. Excessive facet removal was avoided, and the lateral S1 superior articular process was undercut within a safe range to preserve facet continuity. Extensive removal of the sacral ala or the L5 transverse process was generally avoided.

In the LDH group, after nerve root mobilization, the surgeon approached the migrated disc fragment in the extraforaminal direction and removed it using pituitary forceps. When the annular fissure was narrow, careful expansion was performed while minimizing traction on the dura and nerve root.

In the FES group, the primary stenotic components, thickened capsule, fibrous scar tissue, and osteophytes were removed posterolaterally to restore the gliding mobility of the nerve root.

Decompression was considered complete when pulsation of the nerve root, smooth passage of the loop probe, and disappearance of traction-induced electromyographic changes were confirmed. Additionally, an increase of ≥35% in extensor hallucis longus motor evoked potential amplitude compared with baseline was used as an objective indicator of adequate decompression. Small pieces of autologous fat were placed around the nerve root to prevent adhesion. After confirming hemostasis, a suction drain was inserted, and the wound was closed in layers. Postoperative management included standard pain control and early mobilization, with postoperative imaging performed as needed.

Outcome definition

The primary outcome was the achievement rate of the minimal clinically important difference (MCID) in the ODI. MCID achievement was defined as an improvement of ≥13 points from baseline to 12 months postoperatively [11]. ODI scores were calculated using the adjusted score method when unanswered sections were present, with the denominator set as the number of answered sections × 5 [12,13]. Patient-reported outcome measures, including ODI and pain intensity, were collected using self-administered questionnaires during the preoperative and 12-month postoperative follow-up visits. Perioperative indices were extracted from patient medical records. Pain intensity was assessed separately for leg pain and low back pain using an 11-point numeric rating scale (NRS; 0 = no pain, 10 = worst imaginable pain), referring to the average pain over the prior seven days. Changes in ODI and NRS scores were calculated by subtracting the preoperative score from the 12-month postoperative score.

Statistical analysis

Normality of continuous variables was assessed using the Shapiro-Wilk test and visual inspection of histograms. Continuous variables were summarized as medians and interquartile ranges, and groups were compared using the Mann-Whitney U test. Categorical variables were presented as proportions and compared using Fisher’s exact test. The primary analysis compared the achievement rate of ODI MCID between groups, and ΔODI was additionally assessed. A linear regression model adjusted for age and preoperative ODI was constructed, with ΔODI as the dependent variable and group (FES vs. LDH) as the main explanatory variable. All analyses were two-sided, and p < 0.05 was considered statistically significant. Statistical analyses were performed using Stata version 18 (StataCorp, College Station, TX, USA).

## Results

Patient characteristics

During the 60-month study period from January 2015 to December 2019, 129 surgeries were performed for L5/S1 foraminal or extraforaminal lesions. After excluding 45 patients who underwent concomitant surgery at spinal levels other than L5/S1, 84 patients who underwent surgery only at L5/S1 remained. Of these, 32 patients who underwent fusion surgery, one patient with a procedure-related residual deficit, and two patients with missing preoperative ODI data were excluded. Consequently, 49 patients were included in the final analysis, including 27 patients with LDH and 22 patients with FES. Among them, 40 patients completed the 12-month postoperative follow-up and were included in the 12-month outcome analysis (LDH, n = 19; FES, n = 21). Baseline characteristics are summarized in Table 1.

**Table 1:** Baseline characteristics of patients by group (LDH vs. FES) Data are shown as median (interquartile range) or n (%). Statistical significance was set at p < 0.05. Continuous variables were compared using the Mann–Whitney U test; categorical variables were compared using Fisher’s exact test. Mann–Whitney U test z-values: age (z = −1.97), BMI (z = 0.39), operative time (z = −2.09), preoperative NRS back (z = −0.44), preoperative NRS leg (z = −0.45), preoperative ODI (z = 1.41). Fisher’s exact test: sex (p = 0.736), laterality (p = 0.645). *p < 0.05. LDH, lumbar disc herniation; FES, foraminal or extraforaminal stenosis; IQR, interquartile range; ODI, Oswestry Disability Index; NRS, numeric rating scale.

Characteristic	LDH (n=19)	FES (n=21)	p-value
Age, median (IQR) (years)	63.0 (54.0–74.0)	72.0 (67.0–77.0)	0.049*
Sex, n (%)			0.736
Male	14 (73.7%)	14 (66.7%)	
Female	5 (26.3%)	7 (33.3%)	
BMI, median (IQR) (kg/m²)	24.3 (20.6–27.2)	23.7 (21.2–26.4)	0.694
Operative time, median (IQR) (min)	40.0 (29.0–46.0)	49.0 (37.0–59.0)	0.037*
Laterality, n (%)			0.645
Left	13 (68.4%)	13 (61.9%)	
Right	6 (31.6%)	6 (28.6%)	
Bilateral	0 (0.0%)	2 (9.5%)	
Preoperative ODI, median (IQR)	42.0 (31.1–52.0)	33.3 (24.0–46.0)	0.158
Preoperative NRS (back), median (IQR)	5.0 (2.0–7.0)	6.0 (3.0–7.0)	0.661
Preoperative NRS (leg), median (IQR)	5.0 (3.0–8.0)	5.0 (4.0–8.0)	0.652

The median age was significantly higher in the FES group than in the LDH group (72.0 vs. 63.0 years, Mann-Whitney p = 0.049). The proportions of male patients and female patients did not differ significantly between the groups (p = 0.736).

Preoperative pain intensity was comparable between the groups. Low back pain NRS (p = 0.661), leg pain NRS (p = 0.652), and preoperative ODI (LDH, 42.0 (IQR, 31.1 to 52.0) vs. FES, 33.3 (IQR, 24.0 to 46.0); p = 0.158) scores did not differ significantly between the groups.

In contrast, the operative time was significantly longer in the FES group (49 min (IQR, 37 to 59)) than in the LDH group (40 min (IQR, 29 to 46); p = 0.037).

Clinical outcomes

Twelve-month outcomes are summarized in Table 2. The median change in ODI from baseline to 12 months (ΔODI; negative values indicating improvement) was −24.4 (IQR, −36.0 to −10.9) in the LDH group and −17.8 (IQR, −22.2 to −2.2) in the FES group. ODI improvement was greater in the LDH group than in the FES group (Mann-Whitney p = 0.031).

**Table 2:** Twelve-month outcomes by group (LDH vs. FES) Data are shown as median (interquartile range) or n (%). Statistical significance was set at p < 0.05. Continuous variables were compared using the Mann–Whitney U test; categorical variables were compared using Fisher’s exact test. Mann–Whitney U test z-values: ΔODI (z = −2.15), change in NRS back (z = −1.28), change in NRS leg (z = −0.90). Fisher’s exact test: MCID achieved (p = 0.511). *p < 0.05. LDH, lumbar disc herniation; FES, foraminal or extraforaminal stenosis; ODI, Oswestry Disability Index; NRS, numeric rating scale; IQR, interquartile range; MCID, minimal clinically important difference

Outcome	LDH (n=19)	FES (n=21)	p-value
ODI at 12 months, median (IQR)	20.0 (2.2–28.9)	22.2 (0.0–42.2)	—
ΔODI (12 mo–baseline), median (IQR)	−24.4 (−36.0–−10.9)	−17.8 (−22.2–−2.2)	0.031*
Change in NRS (back), median (IQR)	−2.0 (−5.0–0.0)	−0.5 (−4.0–2.0)	0.201
Change in NRS (leg), median (IQR)	−4.0 (−6.0–−1.0)	−3.0 (−5.0–0.0)	0.369
MCID achieved, n (%)	14 (73.7%)	13 (61.9%)	0.511

The achievement rates of MCID (≥13-point improvement) in ODI were 73.7% (14/19) in the LDH group and 61.9% (13/21) in the FES group. The rates were similar, with no significant difference between the groups (Fisher’s p = 0.511).

In the secondary analysis of pain outcomes, the median improvement in low back pain NRS was −2.0 (IQR, −5.0 to 0.0) in the LDH group and −0.5 (IQR, −4.0 to 2.0) in the FES group (p = 0.201). The median improvement in leg pain NRS was −4.0 (IQR, −6.0 to −1.0) in the LDH group and −3.0 (IQR, −5.0 to 0.0) in the FES group (p = 0.369).

A linear regression model adjusted for age and preoperative ODI showed that the FES group had a ΔODI that was, on average, 11.12 points higher than that of the LDH group, indicating less improvement in the FES group (95% CI, −0.15 to 22.39; p = 0.053). The confidence interval included zero, and the estimate should therefore be interpreted with caution (Table 3).

**Table 3:** Linear regression analysis for change in ODI (12-month minus baseline) Multiple linear regression with robust standard errors (F(3, 36) = 3.05, p = 0.041, R² = 0.188). β coefficients represent mean differences in ΔODI; positive values indicate smaller improvement. Statistical significance was set at p < 0.05. Test statistic values: disease (t = 2.00), age (t = −0.47), preoperative ODI (t = −1.56). ODI, Oswestry Disability Index; SE, standard error; CI, confidence interval; FES, foraminal or extraforaminal stenosis; LDH, lumbar disc herniation

Predictor	β (SE)	95% CI	p-value
Disease (FES vs. LDH)	11.12 (5.56)	(−0.15, 22.39)	0.053
Age (years)	−0.09 (0.20)	(−0.50, 0.31)	0.643
Preoperative ODI (score)	−0.26 (0.16)	(−0.59, 0.08)	0.129

## Discussion

In the present study, we strictly classified L5/S1 foraminal or extraforaminal lesions treated with a posterior microendoscopic approach into LDH and FES and compared postoperative outcomes between these two disease entities. While the achievement rates of the ODI MCID were similar between groups, the degree of improvement in ODI was significantly greater in the LDH group. In the adjusted analysis controlling for age and preoperative ODI, the direction of the association was consistent with the unadjusted comparison, although the confidence interval included zero. These findings suggest that LDH may be associated with more favorable functional recovery than FES; however, this interpretation should be made cautiously. 

The surgical treatment of foraminal and extraforaminal lesions at L5/S1 using microscopic or endoscopic posterior approaches has generally shown good outcomes. Previous reports demonstrated significant improvements in pain and functional indices after selective decompression of fibrous or osseous stenosis at the lateral exit zone [14]. Navigation-assisted transtubular decompression also resulted in significant improvements in VAS and ODI scores for L5/S1 foraminal or extraforaminal lesions [15]. Similarly, endoscopic transforaminal or foraminotomy procedures applied to L5/S1 patients have shown favorable short- to mid-term outcomes with low complication rates [7].

However, although classical anatomical descriptions indicate that the L5 nerve root is particularly vulnerable to mechanical entrapment between the transverse process and sacral ala (the far-out region), few studies have compared postoperative outcomes between “mass-effect-dominant LDH” and “fibro-osseous stenosis-dominant FES” at this level [3,8,9]. The present results address this gap by demonstrating greater improvement in functional outcomes (ΔODI) for LDH compared with FES after microendoscopic posterior decompression at L5/S1. One possible explanation is that fragmentectomy in LDH achieves decompression in a relatively localized area, allowing rapid restoration of nerve root mobility and gliding. Conversely, FES often involves fibrous or osseous changes that extend continuously from the foramen to the far-out zone, requiring decompression of a broader area. This may lead to a slower or less pronounced improvement in symptoms and functional recovery.

Preoperative evaluation using standardized MRI, including axial slices and CT, is important for distinguishing the relative contributions of the mass effect and fibro-osseous stenosis. For LDH, a strategy centered on removing the migrated fragment is appropriate, whereas for FES, careful but sufficient management of lateral components, such as the ventrolateral joint capsule, lateral osteophytes, and undercutting of the lateral superior articular process, is required [3,5,6]. Even within the same L5/S1 foraminal to extraforaminal region, the pattern of compression differs markedly between localized disc herniation and continuous fibro-osseous stenosis, making disease-specific planning essential. Prior studies and reports on far-out lesions have also noted that insufficient lateral decompression or overlooked stenotic components can lead to residual symptoms and reoperation [8,16,17]. Our findings are consistent with these prior observations.

This study has several strengths. First, the surgical indications, imaging evaluations, and operative procedures were standardized and performed at a single institution by a single surgeon, minimizing intercase variability and improving internal validity. Second, we limited the analysis to L5/S1 lesions, which are a level with unique anatomical narrowing due to the passage of the L5 nerve root between the transverse process and sacral ala [6,16]. This restriction enhanced the validity of the comparison of disease entities. Third, utilizing intraoperative findings as the final diagnostic criterion reduced the risk of misclassifying borderline cases that can be difficult to distinguish using imaging alone, aligning with the methodology of previous studies by Matsumoto and Lee [3,14]. Fourth, all patients completed a 12-month follow-up, allowing the assessment of clinically meaningful mid-term outcomes under stable conditions. Because L5/S1 foraminal or extraforaminal cases are relatively uncommon compared with central canal lesions, the consistent one-year follow-up adds value.

Nevertheless, this study has several limitations. First, the retrospective design did not eliminate the possibility of residual confounding factors. However, standardized surgical indications and techniques, along with adjusted analyses controlling for age and preoperative ODI, helped minimize this concern. Second, the sample size was relatively small, which may have limited the statistical power. Third, we did not perform quantitative imaging to evaluate stenosis severity or osteophyte formation. Although combining standardized MRI/CT evaluation with intraoperative confirmation helped ensure appropriate disease classification, a more detailed stratification (e.g., foraminal LDH, foraminal stenosis, far-out LDH, and far-out stenosis) may not be feasible because of the frequent continuity of lesions across these zones [16]. Fourth, because this was a single-center study, generalizability may be limited; however, the uniformity of the surgical technique and perioperative management is a practical strength for internal validity. Finally, while the 12-month ODI MCID provided a robust primary functional outcome, longer-term follow-up and comprehensive tracking of recurrence, neurological recovery, reoperation, or adjacent segment disease would enhance the external validity of future studies.

## Conclusions

Microendoscopic posterior decompression for L5/S1 foraminal or extraforaminal lesions resulted in a generally comparable MCID achievement rate between LDH and FES; however, the magnitude of functional improvement (ΔODI) was greater for LDH. These findings suggest that mass-effect-dominant LDH and fibro-osseous-stenosis-dominant FES exhibit different response profiles to the same surgical approach. Early and accurate differentiation of these disease types, along with optimized decompression strategies tailored to each pathology, is essential for maximizing clinical outcomes.
